# The quality of caregiver–child interaction is predicted by (caregivers’ perception of) their child’s interests

**DOI:** 10.1098/rsos.231677

**Published:** 2024-04-24

**Authors:** Rajalakshmi Madhavan, Nivedita Mani

**Affiliations:** ^1^ Psychology of Language Department, University of Göttingen, Göttingen 37073, Germany; ^2^ Leibniz ScienceCampus Primate Cognition, Göttingen 37077, Germany

**Keywords:** category interest, word learning, curiosity-driven learning, word recognition, picture books, shared book reading

## Abstract

This current study examines the extent to which children’s interests and caregivers’ sensitivity to their children’s interests are associated with the quality of caregiver–child interaction, and subsequent learning. Eighty-one caregiver–child dyads (24–30-month old children) completed an online shared book-reading task where caregivers and children read two e-books with pictures and descriptions of objects from different categories—one previously determined to be of low and one of high interest to the child (with one novel word-object mapping introduced in each book). We also obtained separate behavioural indices of children’s interests and children’s later recognition of newly introduced word-object mappings. Our findings highlight that the quality of caregiver–child interaction is predicted by children’s interests and caregivers’ perception of children’s interests, although we find only limited overlap between our behavioural indices of children’s interests and caregiver perception of children’s interests. Neither of these factors predicted later novel word recognition. Thus, while the dynamics between higher quality of caregiver–child interaction, children’s interests and learning remain inconclusive, caregivers and children appear to be more attentive, enthusiastic and engaged in reading about topics that (caregivers believe) interest the child. Furthermore, learning in itself seems to be successful, regardless of factors involved, through the mere task of shared book reading.

## 1. Introduction

Caregivers of young children can usually pinpoint what their child is interested in, be it with regard to their food preferences [1,2] or their interests in art, play and science [3]. Caregivers also engage in scaffolding behaviours that help maintain the child’s interest, like talking about the objects that their child is interested in [4] or organizing materials and enriching activities related to their child’s interests [5,6]. However, caregivers’ efforts to maintain their child’s interests are, obviously, contingent on their being aware of what their child is interested in. While bouts of extremely intense interests in children may be apparent to the caregiver [7], greater sensitivity may be required with regard to their child’s more fleeting short- and long-term interests. Against this background, the current study examines the extent to which the quantity and quality of caregiver–child interaction varies as a function of either children’s interest or children’s perceived interest in the content being explored. Furthermore, given previous studies reporting that children’s interests mediate learning success (e.g. [8–10]), we will examine the relationship between children’s interests, caregiver perception of children’s interests, the quality of caregiver–child interaction and children’s subsequent learning of novel word-object associations introduced in a shared book-reading task. In what follows, we briefly review the literature on the role of caregiver–child interaction and caregiver input, as well as children’s interests in early language development.

### 1.1. The role of the caregiver in shaping early language acquisition

Caregivers undoubtedly play an important role in shaping their child’s learning environment, and consequently knowledge and language acquisition. Sociocultural theories of development, such as Vygotsky [11] or Rogoff [12], emphasize the ‘apprentice-type relationship’ between caregiver and child, where the caregiver guides the child’s participation in their daily activities. By providing them with toys, books and other materials, as well as information about objects and entities in their environment [6], caregivers constitute a critical source of variability in the child’s input, which in turn influences children’s lexical diversity [13,14]. A number of studies showcase the richness of caregiver–child interactions in some societies. High-quality interactions typically include non-verbal cues such as eye gaze, joint attention [15], caregiver-responsiveness to the child [16], the use of infant- or child-directed speech [17,18], co-speech gestures like pointing or manipulating objects [19–21], as well as the use of iconicity (i.e. onomatopoeia and iconic gestures) when the referent is absent from the environment (22, 23). Caregiver–child interactions also present fine-grained child-directed modulations of caregiver actions, termed motionese or infant-directed action (IDA). Similar to IDS [24–26], infants prefer IDA compared with adult-directed action [27]. Modifications for IDA include more frequent eye gazes between the object and the infant (gaze shifts [28,29]), as well as more frequent manipulations and label presentations of the object [19,20].

Many of these attributes of caregiver–child interaction have been linked to later development. Hart and Risley [30] highlighted this in their study, showing that the quantity of caregiver–child interaction—the number of words a child hears—predicts children’s vocabulary growth, while later studies similarly find the quality of caregiver–child speech to be an important factor in early language development [26,31]. Indeed, the degree of caregiver engagement predicts children’s vocabulary scores, from six months to even three years later [32,33]. Maternal responsiveness to infants’ babbling similarly predict both immediate changes to their pre-verbal behaviour and subsequent language milestones [34,35] especially when responsiveness is prompt, contingent and appropriate [36,37].

Such rich interactions or scaffolding events take place in different naturalistic settings, such as joint television viewing [38,39] and shared book reading [40,41]. Indeed, caregiver–child shared reading is an ideal context to foster children’s language abilities, since it serves as a social and a contextual crutch for language development, by engaging both caregiver and child in a conversational interaction where they simultaneously pay attention to a single topic [42]. Shared book reading between caregivers and children is an important activity when it comes to the development of children’s language skills [43,44], and it serves as a means for caregivers to introduce and teach their children new words [45]. Shared book reading helps the parent and child engage in exchanges that can be orderly and predictable [46] and has been shown to boost expressive language skills [47–49], vocabulary growth and later literacy skills ([30,47,50]; see [51] for a meta-analysis), phonological awareness [52,53] and empathy development [54]. The benefits of shared book reading with caregivers have been extended to both analogue and digital contexts, with similar boosts in word comprehension and phonological awareness linked to the duration of time spent reading printed and e-books [55–57]. Meta-analyses have revealed that the caregiver–child shared book reading where the child is an active participant positively impacts their vocabulary development [51].

Caregivers have been shown to modify their verbalizations during shared book reading to the verbal abilities of the child [58], and change their interaction style to match the child’s requirements [59]. Furthermore, caregivers modulate their reading interactions with their children based on their child’s interests. For example, caregivers tend to read more frequently and for longer periods to children who are more interested in books and take more initiatives to begin shared book reading sessions with their child [60,61]. Indeed, caregivers, in general, expose their children more often to activities they are interested in [4], both guiding the interaction and letting the child guide the interaction; engaging in question-and-answer exchanges, and re-engaging them if they show signs of loss of interest [62].

Taken together, the research reviewed in this section highlights the key features of caregiver–child interactions, especially in naturalistic settings like shared book reading, which have been shown to foster language development. Against this background, the current study will examine the extent to which the quantity and quality of caregiver–child interaction varies as a function of caregivers’ perception of their child’s interest in the content being explored and children’s learning from such interactions.

### 1.2. The role of children’s interests in shaping language development

Interest is typically characterized as a predisposition to re-engage with certain content [63]. In young children, interest is typically marked by children keeping certain objects in their visual field or within their reach, and thus ensuring their ability to sustain their interaction with these objects [64,65]. The propensity to re-engage with certain content may develop across time, as characterized by the four-phase model of interest development [63]. Thus, initially, an individual’s interest in an object may be triggered by something in the environment which draws their attention to it, followed by the individual engaging and re-engaging with the object more over time (maintained situational interest). The individual gathers more knowledge about the object without external motivation (emerging individual interest), after which the individual acquires extensive knowledge of the object and prefers to engage with this object over others when given a choice (well-developed individual interest).

Indeed, given the vast amount of verbal and visual information children receive, and basic attentional limitations early in development, children cannot possibly attend to all the input that they are presented with. Piagetian theories of early development suggest that children actively shape their learning experience by selectively sampling from information presented to them [66–68], among others. Children’s interests have been shown to have a powerful influence on knowledge acquisition and perseverance at school, with a meta-analytic coefficient of 0.31 between interest and academic achievement [69]. Earlier in development, children demonstrate their selective interest by actively seeking information about specific objects via pointing [10]. They also retain information provided at their behest better [9,70]. Thus, for instance, when children decide which of many unfamiliar objects they want labelled in a particular order, they later show improved recognition of the object-label associations, relative to when the instructor uses a pre-determined list of objects to name for the child ([71], but also see [72]). They also selectively sample information in situations of ambiguity [73,74], for instance, by selectively eliciting the label of an object when the label of a particular object is ambiguous, relative to when it is more certain [74]. Furthermore, they elicit information from informants who provide more reliable information prospectively and retain information provided from more reliable informants retrospectively [75]. Findings such as these support curiosity-driven theories of learning suggest that children, like ‘little scientists’ [68,76] actively shape their learning experience by selectively sampling from the information presented to them, based on what they want to learn, when they want to learn and whom they want to learn from (see [67] for a review [66]).

While the studies reviewed above highlight the role of missing knowledge or required knowledge in triggering children’s information elicitation, research has also long focused on the role of children’s interests in learning [63,77]. Indeed, even young infants display a propensity to keep certain objects in their field of view and repeatedly re-engage with specific objects in their environment [64]. Recent research using head-mounted images of the infants’ world view suggest that they are selective with regard to what occupies their visual field, by creating situations where only one object from a rich environment is visually dominant, and co-ordinating their visual attention to the object at hand [78]. Later in development, studies suggest that toddlers learn and retain the labels of novel objects better if they are interested in the category the word belonged to, as indexed by parental reports of their children’s interests in—and children’s pupillary arousal to objects from—these categories [8]. Finally, in early education, while children provided with writing support on low-interest topics produced similar quality writing as when writing on high-interest topics, children generated better ideas when writing on high-interest topics [79]. Such findings have led to the development of models of interest development and learning, which highlight the keen association between a child’s interest in a topic, the extent to which this topic is reinforced by the child’s environment and the child’s propensity to re-engage and acquire greater knowledge about this topic [63]. Indeed, caregivers with certain beliefs, for example, with regard to the role of curiosity in learning, and behaviours, for example, ensuring that their child has materials related to their interests at home, have children who show more sustained and long-term interests [6]. Taken together, the research reviewed thus far suggests that children display sustained interests; caregivers may be sensitive to their child’s interests and sustain these interests through certain behaviours; and these interests may play a role in language development. Such individual interest-driven dynamics of caregiver–child interactions may explain reports of differences in the vocabularies of young children observed by Mayor & Plunkett [80], that is, in the words known to individual children, with some infants attending to and learning about some topics and interactions better than others.

### 1.3. Current study

The current study examines the extent to which children’s interest, and caregivers’ perception of their child’s interest in the content being explored in an interaction influences the quality of the caregiver–child interaction and subsequent learning from that interaction. We examine these questions in the context of a naturalistic online shared book-reading task. Reading picture books with young children has been shown to help children in extending newly learned word-object mappings from the book onto real objects [81]; increase toddlers’ willingness to taste and consume familiarized vegetables from the books compared with completely unfamiliar books [82]; and attempt to perform actions to try and elicit novel objects’ non-obvious properties [83]. Considering children of 2–3 years consistently consume screen media often [84], online books or e-books would naturally become options for caregiver–children. E-books, which can be accessed via a multitude of electronic devices such as iPads and tablets, share many traditional characteristics with that of physical or print books, with the added advantage of hosting many other electronic features such as animation or active text [85]. E-books also prompt parents to provide more support and encouragement to their children for interacting with the books, thereby fostering children’s independence [86]. Similar to print book reading, e-book reading is also been shown to successfully enhance children’s language skills [57], engagement during the task [87] and overall parent–child interaction [88]; and in some cases, to be even more impactful relative to regular book reading [89,90].

In the current task, we recorded caregiver–child interaction during shared book reading of a book reported (by the caregiver) to be of high interest to the child and a different book reported (by the caregiver) to be of low interest to the child. The books that caregiver and children were custom-created for this study, with each book also containing a novel word-object mapping that is to be introduced to the child. We coded the quality of interaction (QOI) based on [19,20] measures and modified for the age of the children tested in the current study. In particular, we coded dimensions of attentiveness, interactiveness, enthusiasm and range of motion (see coding for a full breakdown of the measure and its dimensions). We predicted that the QOI would differ according to whether the caregivers reported the book to be of high or low interest to their child (*Hypothesis 1*).

Following the book-reading task, we assessed children’s interest in the six book themes, using a preferential-looking paradigm, where they were shown a number of different objects specific to the six book themes. Thus, in each trial, children were presented with two objects from two different themes, for example, animals and vehicles, as we recorded their looking behaviour to the objects from different themes. We measured the amount of time children spent looking towards objects belonging to the different themes, with longer looking durations indicative of higher preference, and thus interest, in those themes. We predicted an association between children’s looking behaviour towards specific categories and parental ratings of their child’s interests in these categories (*Hypothesis 2*).

Finally, we assessed children’s recognition of the labels for novel objects included in the books, using again the Intermodal Preferential Looking Paradigm. Here, children were presented with two novel objects that they had seen in the low- and high-interest books, respectively, and heard the label for one of these objects. We predicted that children would show improved recognition of the novel label-object associations, that is, look longer at the labelled object, for objects they showed greater interest in relative to objects they showed less interest (*Hypothesis 3*).

## 2. Material and methods

All the data are required to replicate our analyses, outcomes and conclusions that have been published online in an open science repository [91].

### 2.1. Preregistration

We preregistered our sample size (*n* = 70), predictor and response variables, hypotheses and planned analyses on the Open Science Framework (https://osf.io/e83uy) prior to data analysis. The datasets and analysis scripts can be found on the OSF page of the project (https://osf.io/fj4ru/).

### 2.2. Participants

The final sample size for the first analysis consisted of 79 dyads (sample sizes for other analyses are reported below). However, we state that a total of 81 caregiver–child pairs participated in the current study, and not all children provided data for all analyses. Children were German-speaking monolinguals between 24 and 30 months old (*M*_age_ = 26.60, s.d._age_ = 1.84; 42 girls and 39 boys) and were carried to full term with no known developmental disorders. All caregiver–child dyads were recruited from a volunteer database of children managed by the laboratory, and mainly came from families living near and around the city where the university is based. Our rationale for studying children of this age group comes from our previous studies looking at children’s interests and word learning, where we also tested children of the same age group, and found an effect of children’s categorical interests on word-object mapping recognition [72,92]. Additionally, picture book shared-reading studies also often report many benefits of shared book reading for children under 3 years of age, including the acquisition of elaborated word meanings [93] and the capacity of symbolic re-enactment and representations [94]. Twenty-four months old have the capacity to form categories at various levels of generality [95], and also demonstrate an ability to learn novel words belonging to distinct categories by way of leveraging their lexical knowledge in these categories [8,96].

### 2.3. Stimuli

We chose five objects known to be familiar and one object known to be unfamiliar to children at the age tested for each of six categories, namely: animals, clothing, body parts, foodstuff, furniture and vehicles (based on German CHILDES and Wordbank data). We included food items into our stimuli even after considering the issue of the continuous presence of food in children’s daily lives (all children eat), and therefore being of some interest to all children. Food items have been previously used in studies that have examined word learning in 2 year-olds [8,96]. Furthermore, all the categories chosen were taken from the MacArthur Bates Communicative Development Inventory (MBCDI) checklist [97], based on them being likely to be familiar to children of the age tested in the current study. Given limitations on the number of categories likely to be familiar to children at this age, we were constrained in the number of possibilities available to us for use. While the previous studies mentioned above included drinks as a category, however, in the current study, we replaced drinks with furniture, due to increased variability in the physical attributes of furniture relative to drinks. The unfamiliar item (hereafter, the novel item) in each category were as follows: Litschi (Lychee—food), Achsel (armpit—body part), Saiga (saiga—animal), Gugel (gugel—clothing), Vitrine (cabinet—furniture) and Rikscha (Rikscha—vehicle). We chose a further novel item for two categories, in case the child was familiar with the novel object, Guave (guava) for food and Staffelei (easel) for furniture. All nouns shared feminine grammatical gender. Given the novelty of the task with which these novel words were to be introduced to the children (i.e. that caregivers were essentially teaching the child the new words via an online platform), we decided to restrict the number of new words being introduced.

#### 2.3.1. Visual stimuli

Photorealistic images of the novel and familiar objects were obtained from sources such as the Bank of Standardized Stimuli [98] and the Moreno-Martinez & Montoro [99] database. Google images were used when it was not possible to obtain images from these databases. Each image was edited to be identical in size, such that the total pixel count was around 4 00 000 pixels. They were then pasted on a grey background of 1000 × 1000 pixels. All images were also equated for saliency; many of the images had been used in previous peer-reviewed studies, and as such, had already been equated for salience. The images which had not been used previously were equated for salience with the previously used pictures for consistency using the GNU Image Manipulation program (GIMP) [100].

#### 2.3.2. Audio stimuli

Audio stimuli were recorded by a female native German speaker in an infant-directed register. The recorded audio was then normalized for volume and filtered for noise using audacity. Audio prompts in the category interest task included generic sentences such as ‘Was gefällt dir besser?’, ‘Was magst du lieber?’ and ‘Was findest du besser?’ (What do you like more?). Audio prompts in the novel object recognition task were the following two cues: ‘Wo ist der/die/das *X*?’ (Where is the *X*?) and ‘Siehst du den/die/das *X*?’ (Do you see the *X*?), where *X* was the label of one of the images presented onscreen, that is, the target image.

#### 2.3.3. E-books

**Figure 1.**
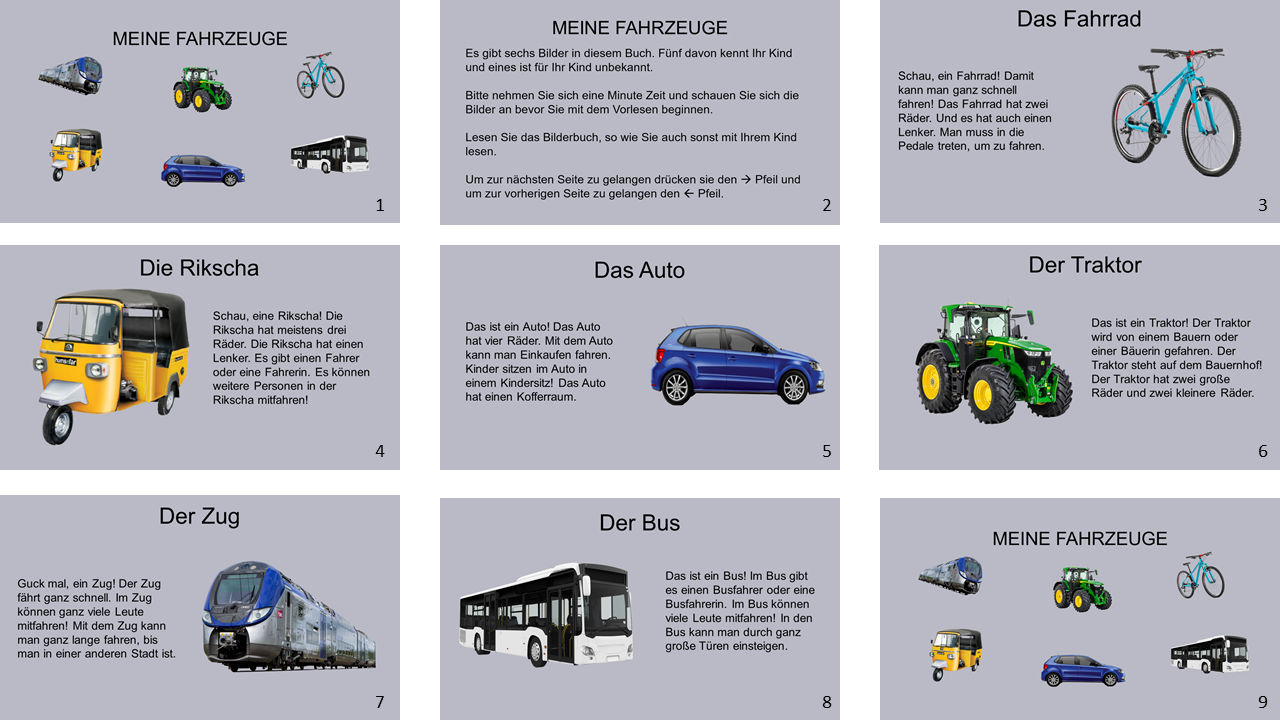
Book on ‘My Vehicles’ comprising five familiar and one novel vehicle object.

We created six different PowerPoint presentations (.pptx file), which served as ‘books’ for each of the six categories. Each ‘book’ comprised nine ‘pages’ and presented children with images of the five familiar and one novel image from that category, against a grey background (figure 1 gives an example of one book). The first and last page contained a collage of all the book objects, and the second page contained an instruction sheet. The rest of the six pages were dedicated to the six objects pertaining to that category. Thus, these pages presented an image of one of the objects for this category and a text related to the object which caregivers could use to guide their interaction. The texts for each object contained approximately the same number of words and no category-specific traits (range: 27–31 words; mean = 28.58; s.d. = 1.32). For the six novel object descriptions, the label of the object was repeated four times in the text. The position of the novel object in each book was pseudo-randomized: the novel object did not appear on the first or the last page.

#### 2.3.4. Caregiver questions

Caregivers were asked to indicate on a scale of 1–7 (1 = not at all, 7 = extremely) how interested their child was in each of the object categories presented (e.g. ‘How interested is your child in vehicles?’). Following this, caregivers were asked whether the child already knew any of the novel objects chosen for the study. We note here that while some caregivers did indicate that children already knew one (or more) of the novel objects already, none of these words were introduced in the books they read (e.g. one caregiver reporting their child already knew the novel food word—lychee—however, the caregiver and child read books about vehicles and furniture during the task). Caregivers were also asked to indicate how often they read with their child, and how they judged the quality of their shared book reading on a scale of 1–7 (1 = child not very engaged, 7 = child very engaged).

### 2.4. Procedure

The study was conducted online using the conference platform ‘BigBlueButton’ (BBB). The platform is hosted on the department server and presented a suitable platform with regard to confidentiality and security issues. Caregivers were contacted by telephone to enquire whether they were interested in participating in the study. If they replied affirmatively, they were asked to answer the questions regarding their child’s interests as described above. Subsequently, caregivers were sent a welcome email with technical specifications and requirements of the study, a data security statement, and the link to the BBB room. Caregivers were asked to confirm on video that they read the data security statement and gave verbal consent for their participation in the study. Next, caregivers were instructed to seat themselves and their child so that their faces were fully visible to the experimenter and their screen was fully visible to them and their child as well. Each dyad had three tasks to complete, in the following order: a shared book-reading task; a category interest task; and, finally, a novel object recognition task. We followed this exact ordering of the tasks, since our critical focus of the study is on the quality of caregiver–child interaction and the extent to which it is predicted by children’s interests and predicted children’s learning. Therefore, children needed to have completed the book-reading task prior to the word learning task. We did not present the category interest task prior to the book-reading task, in case the familiarity with the images in this task influenced our critical measure of the quality of caregiver–child interaction.

**Figure 2.**
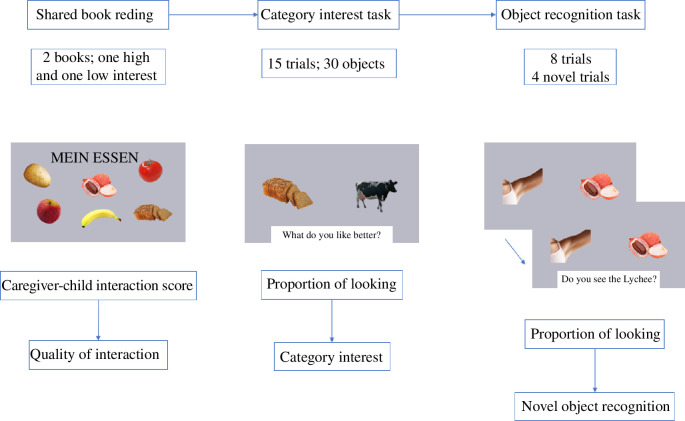
Schematic of the session including ordering and description of tasks.

All sessions were recorded from just before the beginning of the consent statement by the caregivers until the end of the last task. In what follows, we provide more details about each of these tasks (see figure 2 for an overview).

#### 2.4.1. Shared book-reading task

Here, caregivers were given two books to read to their child, one of high interest and one of low interest to the child. The low-interest book was chosen on the basis of the caregiver responses to questions (see §2.3.4 and §2.4), as the book that was rated to be of least interest to the child. For the high-interest book, the tester gave the caregivers a choice between the five category books (excluding the low-interest book), and they were asked to choose a book that they thought would interest their child. The order in which the books were read to the children was counterbalanced. Caregivers had control of the online presentation so they could freely ‘flip’ through the book without hindrance. Caregivers were first asked to familiarize themselves with the objects in the book for about 1 min. During this time, the children were either simply at a different part of the room, or were already sat on the parent’s lap—however, since the familiarization phase lasted only for a few seconds, there was very little possibility for children to have prior exposure to the pictures in the book. Following the familiarization, parents were asked to read the book to their child as they would in a regular reading session with their child. No time limit was given for the reading. However, testers were asked to give parents a gentle reminder that there were more activities to complete if book reading went on for longer than 10 min (this was never the case; all readings lasted less than 10 min—range of reading duration: 1.3–9.96 min).

#### 2.4.2. Category interest task

In this task, children were presented with images of five objects that were already familiar to the children from each of the six object categories, as we recorded their looking behaviour to these objects. Prior to the start of the task, we included a ‘Volume adjustment’ slide, where a short audio clip was played while caregivers adjusted their computer volume to a suitable level. Children were presented with 15 trials containing pairs of images, where the two images in a pair belonged to a different category. Thus, we presented children with 30 images from six different categories (five images per category), such that an item from each category was paired with another item from each of the other categories. The paired objects were first shown on screen for 500 ms, after which the audio cue, ‘Which do you like most?’ was presented. The pictures remained onscreen for 2500 ms after the question ended to give the child enough time to decide between the objects. The length of the auditory stimulus was 2000 ms. Thus, the total trial duration was 5000 ms. A blank screen was shown for 500 ms between trials to signal the end of one trial and the beginning of the next. The trials were grouped into blocks of five, with three trials per block. The ordering of trials was such that all six categories were presented in each block, with no category repeating from the last trial of the previous block to the first trial of the next block. Between every block, an attention-grabbing stimulus was presented for 5 s. We only included an attention grabber every block (i.e. every three trials), since given the fact that we had 15 trials in this task, including an attention grabber between each trial would have unnecessarily increased the duration of the task; which may have led to more children dropping out from the task. Prior studies on children’s interest have similarly used an attention grabber every few trials [8,92]. Since the ordering of objects was pseudo-randomized, this ensured that objects from specific categories rotated across the first and last trials of each block of three trials, thus ensuring that the attention grabber could not have systematically influenced the preferences reported.

During this task, children’s eye fixations during the task were coded offline, and their interest towards each of the categories presented were calculated as the proportion of the amount of time they fixated on the objects belonging to the particular category with respect to their fixations towards all objects from all categories (see §2.4.2 for details). The task took approximately 5 min.

#### 2.4.3. Novel object recognition task

**Figure 3.**
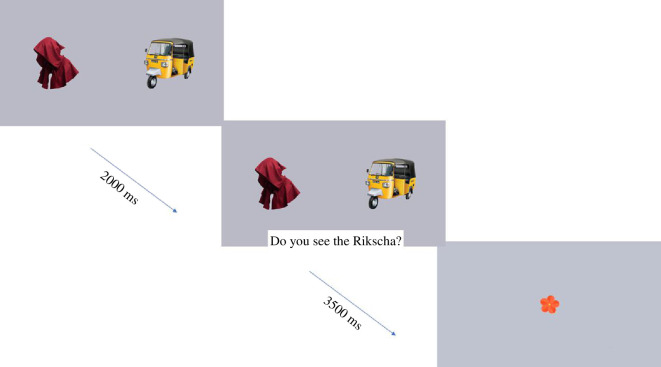
Timeline of a trial in the object recognition task.

The novel object recognition task consisted of eight trials. In four trials, they were tested on their recognition of the two novel objects they learned in the book-reading task (two trials per object), while the other four trials were filler trials, where we examined their recognition of the familiar objects. Each trial presented two images side-by-side on screen, that is, the two novel objects in the novel object trials and two familiar objects in the familiar object trials. The images were presented in silence for 2000 ms, followed by the label for one of the images embedded in a carrier phrase (‘Siehst du den/die/das X?’ or ‘Wo ist der/die/das X?’; see §2.3.2), such that the onset of the label was at 3200 ms. The trial ended at 5500 ms. Between each trial, an attention-grabbing stimulus was played in the centre of the screen (see figure 3). Trial start was contingent on the child attending to the screen, as determined by the experimenter. The ordering of trials and side of presentation of objects were randomized. Children’s eye fixations during the task were coded offline, and their recognition of the novel objects were calculated as the amount of time they fixated towards the target object, divided by their fixations towards both target and distractor object throughout each trial (see §2.5.3 for details). Overall, the task took approximately 2 min.

After the novel object recognition task ended, caregivers were thanked for their time and the session was terminated. They received a thank you letter and an origami crane by post as a token of gratitude.

### 2.5. Data coding

#### 2.5.1. Shared book-reading task

Two blind coders coded the quality of the interaction between caregiver and child in the shared book-reading task. The coders were blind to the goals, hypotheses, procedure and details of the study. They were new research assistants who joined the lab (and therefore had no information about any of the current studies), and were instructed to watch the videos of the book readings and note down the interaction quality ratings. Therefore, they were not aware that the caregiver–child dyads were reading high- or low-interest books, or what analyses would be conducted on the data. The coders were briefed about the details of the study after all the coding for all participants were completed. Videos were coded on four dimensions on a Likert scale from 1 to 6 separately for the caregiver and child, in keeping with Brand *et al.*’s dimensions of IDA [19,20]. The dimensions were (i) attention (how attentive is the child to the book? 1 = not attentive at all, 6 = attention fully on the book); (ii) interactiveness (how often do caregiver and child engage in gaze checking, pointing and representative gestures? 1 = not often at all, 6 = very often); (iii) enthusiasm (separately coded for caregiver and child enthusiasm: how enthusiastic was the caregiver when reading the book to the child/the child when the book was being read to them? 1 = not enthusiastic, 6 = very enthusiastic); and (iv) range of motion (did the caregiver/child reach towards the screen a lot to point at the objects? 1 = very limited range of motion, 6 = a lot of motions). Our final score for QOI was the average of the scores across the different dimensions. For an inter-rater reliability test, we examined the Cronbach’s alpha for the two coders’ ratings of the overall QOI for five different participants (therefore, 10 ratings by each coder; 5 participants × 2 book ratings), which showed high inter-rater reliability (Cronbach’s α value = 0.94).

#### 2.5.2. Category interest task

We manually coded children’s looking behaviour towards the objects presented onscreen using ELAN [101]. We coded whether children were fixating the left or right side of the screen throughout the 5 s of the trial. Gazes were only coded if the look towards the left or right side was longer than 100 ms (or 3 frames). Looks towards the caregiver, to the centre of the screen, and blinks and saccades were not coded. A further coder then checked all ratings for agreement. We calculated the overall duration of fixations towards each object throughout the task, as well as the overall duration of fixations towards each category (sum of fixations to all objects in the category). We then divided the overall duration of fixations per category by the overall duration of fixations to all categories throughout the task. On the one hand, this allowed us to calculate the proportion of time that children looked at objects from the different categories as an index of their varying degrees of interest in the different categories (*child.interest*). This also allowed us to rank the categories based on the proportion of looks to the different categories per participant.

#### 2.5.3. Object recognition task

Here too, we manually coded children’s looking behaviour towards the objects presented onscreen using ELAN [101]. We calculated the proportion of target looking (PTL) within a single trial as the total duration of time the children fixated on the target object, that is, the labelled object, divided by the total duration of time children looked at both the target and distractor across two-time windows. The first time window, the pre-label window, considered all fixations from 0 to 1200 ms after the onset of the test phase, which acted as our baseline measure. The second time window, the post-label window, considered all fixations from 240 ms after the onset of the target label until 2440 ms after the onset (total time window = 2000 ms). We did not perform baseline correction, which was a deviation from our preregistration, and both measures were entered into our statistical model, since we also wanted to determine if children learnt the novel object (and not just whether learning was influenced by various factors).

## 3. Results

All descriptive statistics such as means and standard deviations for all the variables (QOI, duration of reading, looking behaviour of children during the category interest task and proportion of looking towards novel objects during the recognition task) were calculated with respect to caregiver estimates of interest (i.e. high or low interest) and/or by book category. All tables can be found in the electronic supplementary material, S6 [102].

### 3.1. Hypothesis 1: Quality of interactions and caregivers’ estimate of children’s interest in the book

QOI (see §2.4.1 for full coding measures; coded and then averaged as a score between 1 and 6, and rescaled between 0 and 1^1^) was included in the model as the response variable for each of the two books separately. The response was not overdispersed given the model (dispersion parameter 0.53). Caregivers’ estimate of children’s interest in the books was a two-factor variable, high-interest and low-interest books (*caregiver.estimate*). To estimate the extent to which QOI was predicted by the caregiver’s perception of children’s interest in the book, we fitted a Generalized Linear Mixed Model (GLMM [103]; with a beta-error distribution and logit-link function). Apart from our fixed-effects predictor of *caregiver.estimate*, we included a fixed-effects control variable of reading duration (hereafter as *durread*) and a random effects intercept of participant ID in the model. The full model was compared with a reduced model excluding *caregiver.estimate* but including all other fixed and random effects. The sample analysed with this model contained 158 observations of *QOI* from 79 participant dyads.

The results presented in the tables here present estimates of the fixed effects; estimates of random effects for all models can be found in the electronic supplementary material, S5.

**Figure 4.**
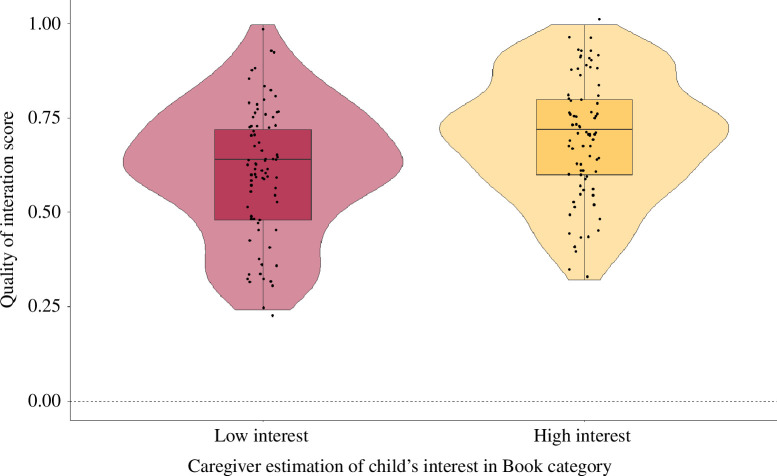
The QOI during book reading varied as a function of caregivers’ estimate of the book being of high or low interest to the child. The black dots show individual observations while the pink and yellow violins depict the distribution density of the response variable according to the two levels of the predictor variable. Horizontal black lines depict the mean of the response according to the two levels of the predictor variable.

The full-null model comparison was significant, *χ* ^2^(1) = 29.98, *p* < 0.001 (table 1 and figure 4), suggesting that the coder’s ratings of the QOI during the shared book reading varied according to caregivers’ estimates of their child’s interest in the book. In particular, the QOI during the shared book-reading task was coded as being higher when reading books caregivers rated as being of high interest to their child relative to books they rated as being of low interest to their child.

**Table 1.** Results of the GLMM examining QOI and *caregiver.estimate*.

	QOI			
predictors	estimates	s.e.	CI	*p*
(intercept)	0.543	0.092	0.378–0.733	
*caregiver.estimate*	0.380	0.063	0.262–0.503	0.000*
*durread*	0.236	0.061	0.116–0.357	0.000*

The predictor durread was *z*-transformed to a mean of 0 and s.d. of 1; mean and s.d. of the original predictor were 3.48 and 1.73, respectively. Indicated are estimates, together with s.e. and 95% CIs. Numbers with* indicate significant p-values; numbers in bold with * indicate significant p-values relevant to interpretation of model (*p* < 0.05).

In addition to our preregistered analysis of the QOI (as reported above), we also examined whether the QOI varied as a function of caregivers’ ratings of their child’s interest in the object book categories (the ratings we obtained from the caregiver questions prior to the study appointment). As mentioned in §2.3.4, we asked caregivers to rate how interested their child was in each of the six object categories presented on a scale of 1–7. To estimate the extent to which QOI was predicted by caregivers’ category interest ratings (hereafter *caregiver.rating*), we fitted a GLMM with a beta-error distribution and logit-link function. In this model, we included our fixed-effects predictor of caregiver ratings of children's interests, along with our control variable of reading duration (*durread*), and a random effects intercept of participant ID. The full model was compared with a reduced model excluding *caregiver.rating* but including all other fixed and random effects. The sample analysed with this model contained 158 observations of *QOI* from 79 participant dyads. We also checked for overdispersion, which is when the variance in the response variable is larger than implied by the respective error distribution (in this case, the beta-error distribution). That is, if our response is overdispersed (given the fitted model), it means that the response has too many values with a large deviation from the mean. Our response was not overdispersed given the model (dispersion parameter 0.53).

**Figure 5.**
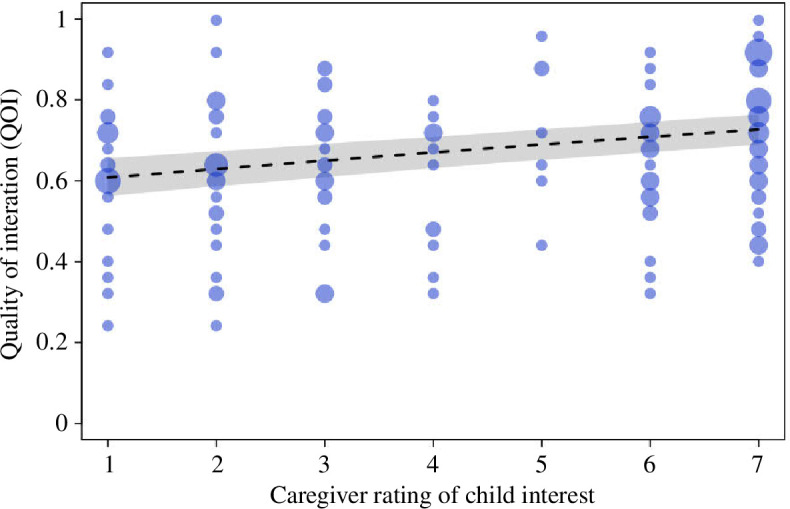
The QOI during book reading varied as a function of caregivers’ rating of the book categories (on a scale of 1–7). Dots show observations whereby the area of the dots (i.e. how big the dot is/how much space the dot occupies on the graph) depicts the number of observations with the exact same rating in both variables (range: 1–7). The dashed line and grey polygon depict the fitted model and its 95% confidence limits. For readers wishing to see a different visualization of data, we have provided plots where each individual data point is represented by a single dot, for ease of interpretation in the electronic supplementary material,** S7**.

The full-null model comparison was significant, *χ* ^2^(1) = 33.51, *p* < 0.001 (table 2 and figure 5), suggesting that the coder’s ratings of the QOI during the shared book reading varied according to caregivers’ ratings of their child’s interest in the book. Specifically, the QOI during the shared book-reading task was coded as being higher when reading books caregivers rated as being of high interest to their child relative to books they rated as being of low interest to their child.

**Table 2.** Results of the GLMM examining QOI and *caregiver.rating*.

	QOI			
predictors	estimates	s.e.	CI	*p*
(intercept)	0.735	0.088	0.565–0.315	
*caregiver.rating*	0.205	0.032	0.145–0.269	0.000*
*durread*	0.247	0.060	0.118–0.367	0.000*

The predictor *caregiver.rating* and *durread* was *z*-transformed to a mean of 0 and s.d. of 1; mean and s.d. of the original predictors were 4.30, 2.28 and 3.48, 1.73, respectively. Indicated are estimates, together with s.e. and 95% CI. Numbers with* indicate significant p-values; numbers in bold with * indicate significant p-values relevant to interpretation of model (*p* < 0.05).

We also conducted another exploratory analysis examining the relationship between children’s interest in the objects presented in the book (as indexed by looking behaviour during the category interest task, calculated as the proportion of time children spent looking at each object category: hereafter *child.interest*) and the QOI during shared book reading of books containing the same objects. To this end, we fitted a GLMM with a beta-error structure and logit-link function, with *QOI* as the response variable, and *child.interest* as the predictor and *durread* as a control variable. We also included the random effects intercept of participant ID in the model. The response was not overdispersed given the model (dispersion parameter 0.58). The sample analysed with this model contained 140 observations of *QOI* from 70 participant dyads.

**Figure 6.**
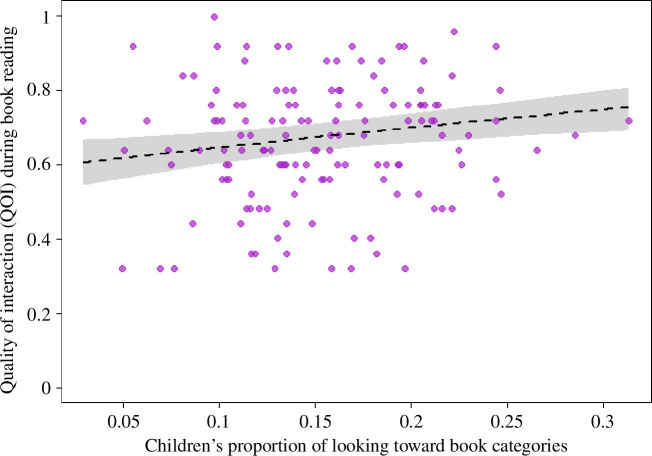
The QOI during book reading as a function of children’s interest in the objects in the book (as indexed by looking behaviour during the category interest task). The dashed line and grey polygon depict a fitted model and its 95% confidence limits.

As with caregivers’ estimate of children’s interest in the different books (*caregiver.estimate*), the QOI varied as a function of *child.interest*; that is, the proportion of time children spent looking at specific objects during the category interest task was positively associated with the quality of caregiver–child interaction when reading books containing those objects (*χ* ^2^(1) = 8.29, *p* = 0.004; table 3 and figure 6).

**Table 3.** Results of the GLMM examining QOI and *child.interest*.

	QOI			
predictors	estimates	s.e.	CI	*p*
(intercept)	0.730	0.083	0.565–0.887	
*child.interest*	0.124	0.042	0.048–0.202	0.004*
*durread*	0.321	0.064	0.192–0.451	0.000*

The predictors were *z*-transformed to a mean of 0 and s.d. of 1; mean and s.d. of *child.interest*: 0.15 and 0.05; *durread*: 3.47 and 1.64, respectively. Indicated are estimates, together with s.e. and 95% CI. Numbers with* indicate significant p-values; numbers in bold with * indicate significant p-values relevant to interpretation of model (*p* < 0.05).

### 3.2. Hypothesis 2: Association between caregiver estimates of children’s interests and children’s looking behaviour

As described in §2.4.2, we computed the proportion of time children spent looking at each object category (time looking at objects from category X/time spent looking at all objects). This allowed us to rank categories (1 was the category children spent most time looking at and 6 was the category children spent the least amount of time looking at in the category interest task). Next, we calculated the number of caregivers whose choice of the high-interest book overlapped with the book ranked highest in the category ranking, that is, the book containing objects children spent most time looking at (in the category interest task). Since looking time could be a noisy measure (i.e. there could be only small differences between the looking time towards the most looked category versus the second most looked category^1^), we also calculated the number of caregivers whose choice of the high-interest book overlapped with one of the top three books in the category ranking. The sample analysed here contained 70 caregiver estimates of the high-interest book and 420 category rankings for 70 participant dyads.

**Figure 7.**
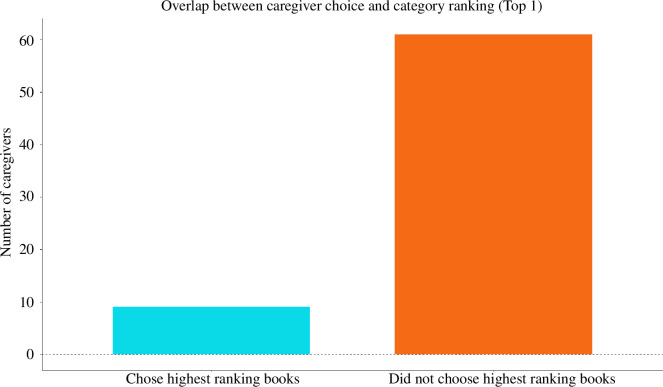
Number of caregivers who chose the book containing objects children spent most time looking at (in the category interest task) as the high-interest book in the shared book-reading task.

**Figure 8.**
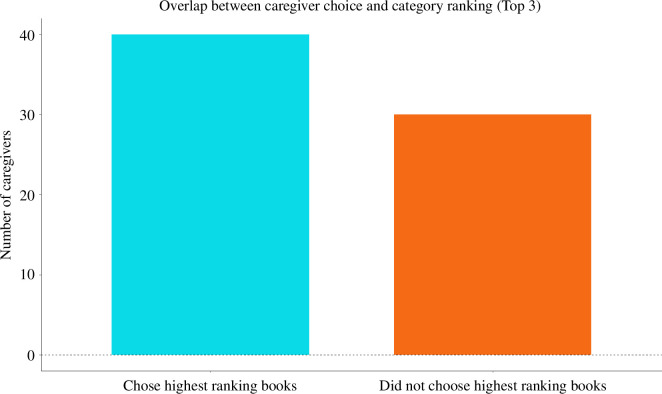
Number of caregivers who chose one of the top three books containing objects children spent most time looking at (during the category interest task) in the shared book-reading task.

Only 12.86% (9 out of 70 dyads) of caregivers chose the highest-ranking book, that is, the book containing objects from the category children spent most time looking at in the category interest task, as the high-interest book (figure 7). Furthermore, 57.14% of caregivers chose one of the top 3 categories in the category ranking as the high-interest book (40 out of 70 dyads; figure 8).

As the caregiver choice of high- and low-interest books is not a continuous measure (with only two choices made by the caregivers overall), we also conducted an exploratory analysis, where we examined the association between caregivers’ ratings of their child’s interest in the object book categories and children’s proportion of time spent looking at each object category. To this end, we fitted a GLMM with a beta-error structure and logit link function. Our response variable was the caregiver’s ratings of interest, while we included the children’s looking time towards objects as the index of interest as the sole fixed-effects predictor. We also included a random effects intercept of participant ID. We compared this model to a null model that included only the random effects. The sample analysed with this model comprised 420 observations from 70 participants.

**Figure 9.**
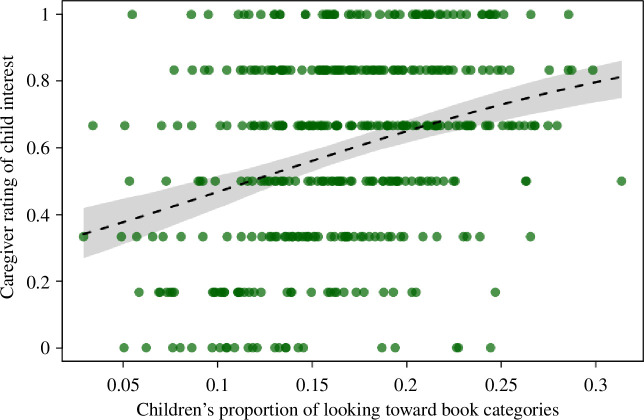
Caregivers’ rating of how interested their child was in each of the categories presented as a function of children’s interest in the objects in the book (as indexed by looking behaviour during the category interest task). The dashed line and grey polygon depict a fitted model and its 95% confidence limits.

The full-null model comparison was significant, and as with the other models, the proportion of time children spent looking at specific objects during the category interest task (*child.interest*) was positively associated with caregivers’ ratings of children’s interests in the range of book categories prior to the shared book reading (*caregiver.rating*) (*χ* ^2^(1) = 39.58, *p* < 0.001; table 4 and figure 9).

**Table 4.** Results of the GLMM examining *caregiver.rating* and *child.interest*.

	*caregiver.rating*			
predictors	estimates	s.e.	CI	*p*
(intercept)	0.368	0.060	0.249–0.487	0.000*
*child.interest*	0.387	0.061	0.270–0.502	0.000*

The predictor *child.interest* was z-transformed to a mean of zero and standard deviation (sd) of one; orignal mean 0.17 and sd 0.05. Numbers with* indicate significant p-values; numbers in bold with * indicate significant p-values relevant to interpretation of model (*p* < 0.05).

### 3.3. Hypothesis 3: Novel object recognition as a function of QOI and children’s interest in the categories

The proportion of looking towards the labelled objects (time spent looking at the target object relative to all objects on screen; separately calculated for the pre- and post-label window, see §2.5.3 for details; *PTL*) was entered as the response variable in this analysis. To examine the extent to which children’s recognition of novel objects (*PTL*) was influenced by the QOI and children’s interest in the books, we fitted a GLMM with beta-error distribution and logit-link function. Our fixed-effects predictors were *QOI*, *child.interest* (proportion of time children spent looking at objects from different categories during the category interest task) along with the control variable of *durread*, and time window within trial (pre- or post-label, hereafter referred to as *window*) on all fixed-effects terms. We added random effects intercept of participant ID, Trial ID, target object and random slopes of *window* and *durread* by the target object in the trial. We removed all parameters for correlations among the random slopes, since the model including the correlations did not converge. We compared this full model with a reduced model including only the control variable of *durread* and all random effects. The response was not overdispersed given the model (dispersion parameter 0.88). The sample analysed with this model contained 482 observations for 67 participants.

**Figure 10.**
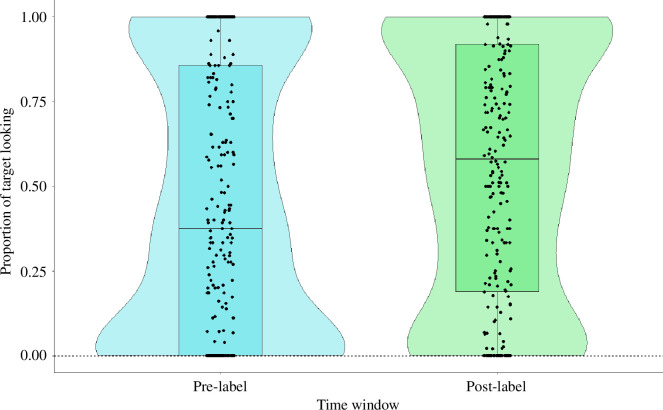
Proportion of looking towards the target object (PTL) as a function of time window (pre-label versus post-label), showing that children learned and recognized the novel word-object associations. The black dots show individual observations while the blue and green violins depict the distribution density of the response variable according to the two levels of the predictor variable. Horizontal black lines depict the mean of the response according to the two levels of the predictor variable.

There was no significant difference between the full and reduced models (*χ* ^2^(6) = 8.55, *p* = 0.20). In other words, there was no association between children’s recognition of the objects in the novel word-object recognition task (PTL) and the QOI during shared book reading (where children were introduced to the novel word-object associations) or children’s interest in the objects presented in the books (as estimated by the proportion of time children spent looking at these objects in the category interest task, *child.interest*). However, there was a significant positive effect of window (table 5 and figure 10), suggesting that children looked longer at the target object in the post-label window relative to the pre-label window, that is, that they had learned the word-object associations. Further drop1 analysis cycled through the individual predictors, excluding them one at a time, and found no significant interaction effects between the predictors. Given that there were no significant interactions between window and other fixed-effects predictors, we built a reduced model excluding this interaction but retaining all fixed-effects predictors (including the main effect of window), which also showed a significant positive main effect of window on PTL (electronic supplementary material, file S1).

**Table 5.** Results of the first GLMM examining object recognition.

	PTL					
predictors	estimates	s.e.	CI	*χ* ^2^	d.f.	*p*
(intercept)	−0.177	0.197	−0.561 to 0.208			
*child.interest*	0.065	0.093	−0.108 to 0.243			0.480
*QOI*	−0.072	0.095	−0.245 to 0.111			0.445
*durread*	−0.081	0.111	−0.300 to 0.129			0.470
*window*	0.302	0.140	0.057 to 0.555			0.032*
window * child.interest	0.049	0.129	−0.171 to 0.279	0.146	1	0.701
window * QOI	0.090	0.127	−0.161 to 0.343	0.501	1	0.479
window *durread	0.097	0.130	−0.158 to 0.344	0.558	1	0.456

All the predictors were *z*-transformed to a mean of 0 and s.d. of 1; mean and s.d. of *child.interest*: 0.15 and 0.05; QOI: 0.65 and 0.17; *durread*: 3.51 and 1.68, respectively. Indicated are estimates, together with s.e., 95% CI and significance tests. *p*-values are derived from the model summary while the *χ* ^2^ and d.f. values are derived from the drop1 results. Numbers with* indicate significant p-values; numbers in bold with * indicate significant p-values relevant to interpretation of model (*p* < 0.05).

Our first exploratory analysis added the fixed effect of *caregiver.estimate* (caregivers’ estimate of whether the book was of high or low interest to the child) to the model above. We included random effects intercepts of participant ID, trial ID and target object, and random slopes of *durread, child.interest, QOI, parent.estimate* and *window* by participant ID and *durread* and *window* by target object, and removed all parameters for correlations among random slopes due to convergence issues. We compared this model to a reduced model including only the control variable of *durread* and all random effects. The response was not overdispersed given the model (dispersion parameter 0.82). The sample analysed with this model contained 482 observations for 67 participants.

**Figure 11.**
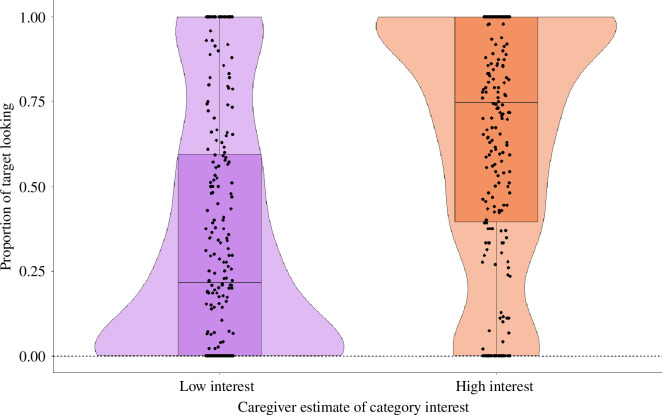
Proportion of looking towards the target object (PTL) across both pre- and post-label windows as a function of caregivers’ estimate of their child’s interest in the book introducing the target object to the child. The black dots show individual observations while the purple and orange violins depict the distribution density of the response variable according to the two levels of the predictor variable. Horizontal black lines depict the mean of the response according to the two levels of the predictor variable.

There was a significant difference between the full and reduced model (*χ* ^2^(8) = 21.76, *p* = 0.005). The model results showed that there was no association between PTL and QOI or *child.interest* as in the model above. However, there was a significant positive effect of *caregiver.estimate* (table 6 and figure 11), that is, caregiver’s estimate of whether the book containing the novel word-object associations was of high or low interest to the child, on children’s attention to the novel objects (PTL). Further drop1 analysis cycled through the individual predictors, excluding them one at a time, and found no significant interaction effects between the predictors. In other words, *caregiver.estimate* did not interact with window, that is, there was no effect of caregivers’ estimate of their child’s interest in the book on children’s learning and recognition of the novel word-object associations in the book. The main effect of *caregiver.estimate* suggests that children across the pre- and the post-label window looked more towards the novel object presented in the book estimated by caregivers as being of high interest to their child relative to the book estimated to be of low interest to the child. Given that there were no significant interactions between window and the other fixed-effects predictors, we built a reduced model excluding this interaction but retaining all fixed-effects predictors (including the main effect of window), which showed a significant positive main effect of window and *caregiver.estimate* on PTL (electronic supplementary material, file S2).

**Table 6.** Results of the second GLMM examining object recognition.

	PTL					
predictors	estimates	s.e.	CI	*χ* ^2^	d.f.	*p*
(intercept)	−0.571	0.142	−0.819 to −0.275			
*caregiver.estimate*	0.792	0.223	0.311 to 1.187			0.000*
*child.interest*	0.086	0.099	−0.109 to 0.281			0.384
*QOI*	−0.067	0.094	−0.241 to 0.119			0.475
*durread*	−0.076	0.095	−0.263 to 0.112			0.420
*window*	0.095	0.177	−0.267 to 0.433			0.589
window*caregiver.estimate	0.426	0.263	−0.112 to 0.933	2.572	1	0.105
window*child.interest	−0.028	0.127	−0.283 to 0.216	0.048	1	0.827
window*QOI	0.066	0.128	−0.187 to 0.336	0.267	1	0.605
window*durread	0.100	0.126	−0.139 to 0.370	0.630	1	0.428

All the predictors were *z*-transformed to a mean of 0 and s.d. of 1; mean and s.d. of *child.interest*: 0.15 and 0.05; QOI: 0.65 and 0.17; *durread*: 3.51 and 1.68, respectively. Indicated are estimates, together with s.e., 95% CI and significance tests. *p*-Values are derived from the model summary while the *χ* ^2^ and d.f. values are derived from the drop1 results. Numbers with* indicate significant p-values; numbers in bold with * indicate significant p-values relevant to interpretation of model (*p* < 0.05).

The second exploratory analysis added the fixed effect of *caregiver.rating* (caregivers’ ratings on a scale of 1–7 on how interested their child was in the categories) to Model 3. We included random effects intercepts of participant ID, trial ID and target object, and random slopes of *durread, child.interest, QOI, parent.rating* and *window* by participant ID and *durread* and *window* by target object, and removed all parameters for correlations among random slopes due to convergence issues. We compared this model to a reduced model including only the control variable of *durread* and all random effects. The response was not overdispersed given the model (dispersion parameter 0.84). The sample analysed with this model contained 482 observations for 67 participants.

We again found a significant difference between the full and reduced model (*χ* ^2^(8) = 22.02, *p* = 0.005). The results from the previous models (models 3 and 4) were also replicated here, that is, we found no significant effect of *child.interest* or QOI on PTL, nor any interaction effects between the predictors. We again found a main effect of *caregiver.rating* and *window*, which suggests that children across the pre- and the post-label *window* looked more towards the novel object when parents rated that object category of more interest to the child; and that children looked longer towards the target object in the post-label *window* relative to the pre-label *window*, indexing successful learning (electronic supplementary material, table S7).

In further exploratory analyses, we substituted within our analysis model the predictor QOI with each of its five individual dimensions (i.e. attention, interactiveness, caregiver, child enthusiasm and range of motion; see §2.4.1). In all of these models, we found no difference between the model estimates and significance values of these five models and our original models with QOI as predictor. Therefore, these models are not any more informative of word learning than our original models. We report these tables in electronic supplementary material, tables S8 and S9.

## 4. Discussion

The current study examined the extent to which children’s interests and caregivers’ perceptions of their children’s interests influence the quality of caregiver–child interaction and children’s learning of novel word-object associations from such interactions. In brief, we found evidence that the quality of caregiver–child interaction was predicted by caregivers’ perception of their children’s interests, and on further examination, children’s behavioural indices of interest. Our examination of the overlap between children’s behavioural indices of interest and caregivers’ reports of children’s interests yielded mixed results. On the one hand, when we compared caregivers’ choice of high-interest books with the categories that children looked the longest at, we found no overlap. However, when considering a more continuous measure, we found a positive association between caregivers’ ratings of their child’s interest in different categories and the amount of time children spent looking at objects from these categories. Novel word-object learning and recognition were not affected by either children’s interests, caregiver perception of children’s interests or the quality of caregiver–child interaction. However, during the novel word-object recognition task, children overall looked more at the novel objects from the high-interest book (as reported by the caregiver) relative to the low-interest book. In what follows, we will first discuss the association between the QOI and caregivers’ perception of children’s interests, followed by the exploratory analyses on the association between the QOI and children’s interests, as indexed by children’s looking behaviour in the category interest task.

### 4.1.Quality of caregiver–child interaction, children’s interests and caregiver perception of children’s interests

Our index of QOI was a composite measure of the attentiveness, interactivity and enthusiasm of both caregiver and child. Our preregistered analyses found that this QOI score was predicted by caregivers’ perception of their child’s interest in the content being explored. Thus, coders rated the interaction as being of higher quality, that is, rated caregiver and child attentiveness, interactivity and enthusiasm higher, when caregivers perceived the book as being of high interest to their child relative to when they rated the book as being of low interest to their child. This pattern of results was replicated, when we used caregivers’ individual interest ratings for each book category to predict the QOIs; with increasing magnitude of caregiver ratings of children’s interest in the book categories, coders also rated the interaction quality to be higher. Separate exploratory analyses found that this pattern of results was consistent across the individual metrics that were entered into the composite QOI score (electronic supplementary material, table S3).

Thus, regardless of whether the QOI metric focused on the caregivers’ behaviour or the child’s behaviour during the interaction, caregiver perception of children’s interest in the content of the book predicted how engaged, enthusiastic and attentive both caregiver and child were. This is consistent with previous research showing that caregiver perceptions of their children has an effect on the quality of caregiver–child interaction, for example, caregivers’ perception of children’s readiness to read modulates the QOI during book reading [104]. Here, we see that caregivers’ beliefs about how much their child will enjoy the book modulates the QOI. We also see that, in line with previous research, children as emergent readers have preferences as to what type of book they would like to read with their caregivers [105].

On the one hand, this pattern of results could be explained by feedback loops between caregiver and child. Thus, the results could be taken to suggest that caregivers were able to accurately estimate their child’s interest in a topic, such that their child was more enthusiastic when being presented with content of interest. Furthermore, against the background of studies suggesting that children’s positive states are generally mirrored by the caregivers [62,106], caregiver enthusiasm in reading about a topic of interest to their child may mirror their child’s engagement. This would lead to the caregiver being more attentive and enthusiastic during the interaction, potentially contributing more to the topic of conversation, which may boost their overall interactiveness and interaction quality during the book reading. This would suggest that caregivers may be able to accurately estimate their child’s interests and that their beliefs about what their child is interested in may improve the QOI between caregiver and child.

Further evidence in favour of the above explanation is provided by our exploratory analyses finding that coders also rated the interaction as being of higher quality the longer children had spent looking at the objects in the book during a separate later category interest task: looking behaviour during this category interest task was our behavioural index of children’s interest in the different categories. Thus, our analysis suggests an association between children’s interests, as measured by this more child-focused measure of interest, and the quality of caregiver–child interaction in the shared book-reading task. This result suggests that caregivers were able to accurately estimate what their child was interested in, in that children were more engaged and attentive to objects from categories that parents indicated being of high interest to the child. Furthermore, the effect of interest on the QOI was consistent across the different measures of interest, that is, caregivers’ ratings of their child’s interest in the categories or children’s looking durations towards objects from these categories were both associated with the QOI during shared book reading.

An alternative interpretation of our results is that the higher quality of caregiver–child interaction in reading a high-interest book may be caregiver-driven, that is, driven by caregivers’ belief that their child is interested in the topic leading to them being more engaged in the book and eliciting their child’s attention to the content and triggering their child’s enthusiasm in the interaction. This may be the case regardless of whether the book was of high or low interest to the child. It may also be due to the fact that caregivers were asked to select a book that would be of high interest to their child, just before reading the chosen high-interest book. However, our findings noted above speak against such an explanation. Specifically, we note that we also found an association between the amount of time children spent looking at objects from specific categories and caregiver–child interaction quality. This suggests an association between children’s interest in the topic of the book, caregivers’ perception of children’s interest in the book and the quality of caregiver–child interaction during shared book reading.

### 4.2. Overlap between children’s interests and caregiver perception of children’s interests

We found mixed evidence of overlap between children’s behavioural indices of interest and caregiver reports of their children’s interest. First, we examined the proportion of time children spent looking at objects from different categories and ranked categories according to the decreasing proportion of looking. We found that caregivers rarely chose to read the book containing objects from their child’s top-ranked category (9 out of 70). Indeed, only 57.14% of caregivers chose to read a book containing objects from one of the top three categories (chance = 60%), as indicated by their child’s looking behaviour (40 out of 70). This discrepancy is mirrored in previous work from our lab showing that caregiver reports and children’s behavioural indices of interest are not associated with one another [107]. Indeed, anecdotally, neither did children’s explicit choice of the book they wanted to read correspond with their looking behaviour. In particular, out of the 70 dyads, six caregivers asked the child to choose the high-interest category book. None of these six children chose the category ranked highest (or top 3 highest) according to their looking behaviour during the category interest task. While this sample is too small to allow firm conclusions, this pattern of results is in keeping with previous work suggesting that children’s explicit choice does not appear to be associated with their implicit-looking behaviour [107]. On the one hand, this may suggest that caregivers may not actually be attuned to what their child is interested in, a suggestion that has implications for the other research questions being addressed here. On the other hand, as is argued in other work [107], our behavioural index of children’s interest may not capture the same construct of interest as captured by caregivers’ reports [63]. Recently, examination of children’s looking behaviour measures have been shown to have little reliability [108], suggesting that looking behaviour may not measure our construct of interest. Alternatively, children’s looking behaviour in the category interest task may be tapping into more fleeting situational interest or arousal towards a particular object relative to another. In contrast, caregivers’ perception of children’s interests and the QOI score may reflect more long-term interests, potentially benefitting from a feedback loop between caregiver perception of interests, and caregiver and child enthusiasm and engagement.

However, exploratory analysis found that caregivers’ ratings of the six object categories in terms of children’s interests in them were positively associated with children’s looking behaviour. In particular, when we consider caregivers’ ratings of their child’s interest in specific categories rather than their choice of high-interest book, children’s looking behaviour successfully predicted caregiver ratings (electronic supplementary material, table S4). Thus, when parents were allowed to rate the categories rather than choose a single book of high interest to read, there appeared to be more consistency across caregiver ratings and children’s looking time behaviour. This exploratory analysis, therefore, suggests greater overlap between children’s interests as estimated by their looking behaviour and caregivers’ estimates of their child’s interests and requires verification in further studies.

Taken together, while certain measures of interest (here, caregiver rating of children’s interest) seem to converge with other measures (here, children’s preferential-looking behaviour as an index of interest), others do not (caregiver choices of high- and low-interest books). Specifically, the caregivers’ dichotomous ‘high’ or ‘low’ interest measure does not relate to either children’s looking behaviour or their own ratings. This may be further taken to suggest that interest is gradient—children are not simply interested or disinterested in certain objects, categories or topics, but indeed, have varying amounts or levels of interest and propensity to engage with them.

### 4.3. Novel word-object recognition and children’s interests

While we found robust evidence that children learned and recognized the novel word-object associations introduced to them in the shared book-reading task, there was no evidence that novel word-object recognition (as indexed by the proportion of looking towards the target object) varied as a function of the QOI during book reading or children’s interests (as indexed by their looking behaviour in the category interest task). While the exploratory analysis found that caregivers’ perception of children’s interests (quantified in two distinct ways, one as the caregiver choice of book, other as caregiver rating of children’s interest in the object book categories) predicted children’s looking behaviour throughout the trial, there was no interaction between time window and caregivers’ estimate of children’s interests, that is, learning and recognition were not impacted by the latter.

In all of these analyses, we found a significant effect of window of analysis, that is, children looked more towards the target object after the label was heard (post-label), compared with before the label was heard (pre-label). The increased looking towards the target object after label presentation suggests that children successfully mapped the target word to the object on the screen, showing that children recognized the object that they were introduced to when they read the books with their caregiver. This is in line with previous research about book reading and vocabulary development. Several studies have shown the link between these caregiver–child shared book reading at home have been shown to successfully predict later reading achievement [109], meaning-related-talk [110] and expressive language and school readiness [111].

However, there may be other factors that may explain children’s looking behaviour towards the target object. For example, since there was only one novel word introduced in each book, it highlighted these novel objects to the children and made them more memorable, resulting in children successfully recognizing both the novel objects in the recognition task. Second, while this paradigm has been extensively used in many eye-tracking studies as an index of children’s recognition [8,112,113], one cannot rule out the effects of mutual exclusivity. In other words, children may have successfully learnt only one novel word-object mapping. When they then see their now-familiarized object and the not-yet-familiar object on screen and hear the label that does not map onto their now-familiarized object, they may look to the other object on the screen, reasoning that their now-familiar object cannot have two labels at the same time. To address this confound, we conducted a simple correlation test to see whether the PTL for high-interest items was correlated with the PTL for low-interest items, which would be the case for a mutual exclusivity-based strategy. We found no evidence for such a correlation. While this suggests that children were not using a mutual exclusivity-based strategy, that is, using their knowledge of only the high-interest category object to infer the mapping of the low-interest category object, we acknowledge that we cannot draw strong conclusions about this confound.

The main effect of caregiver estimates of children’s interests suggests that, overall, children looked more towards the novel object from the high-interest book relative to the low-interest novel book (see figure 10). This may indicate children’s propensity towards engaging with the novel object from the high-interest book—they show increased attention towards this novel object. This cannot be explained by the visual or emotional salience of the object, since different children had different high-interest novel objects, and all of the objects were introduced to them for the first time only a few minutes before the recognition task. Thus, the results from the novel word-object recognition task suggest that learning was relatively unimpacted by the quality of caregiver–child interaction, children’s behavioural indices of interest (from the category interest task) and caregivers’ perception of children’s interests. However, the finding that children overwhelmingly fixated on the novel object from the high-interest book (as indicated by the caregiver) underscores some degree of overlap between caregivers’ perception of children’s interests and children’s looking preferences and corroborates our earlier finding of an association between caregivers’ ratings of their children’s interest in the book categories and children’s looking behaviour.

This study is not without its limitations. Since the study involved three tasks and each session lasted approximately 20 min altogether, the sequence of tasks was not counterbalanced, that is, the shared book-reading task was always the first task the caregiver–child dyads performed, followed by the category interest task, and finally, the novel object recognition task. The sequence of the tasks may have had an effect on the behaviour of the children. For example, since the category interest task always followed the book reading, the QOI during the book reading may have primed children to look more towards the objects from the caregiver’s choice of the high-interest book category, due to the positive feedback loop between the high-interest book choice and the QOI. Additionally, while we tried to examine the role of mutual exclusivity in the novel object recognition task, it may still be possible that children may have successfully learnt only one novel object-word association (most likely one that is of high interest to them) to disambiguate the label of the other novel object on the screen. Future studies can address these limitations, as well as examine other features of real-time interaction, such as introducing more novel words in the picture books to add greater power and variability to the data; and also investigate social looks towards conversation partners [114,115] as a way for caregivers and children to establish common ground and assess behaviours and responses of their interactive partners [116–118].

Taken together, our results suggest that both children’s interests and caregivers’ perception of children’s interests impact the quality of caregiver–child interaction, showcasing the role of interest in driving the QOI between children and their caregivers. As noted above, this could trigger feedback loops in both directions, with both children’s real interest eliciting caregiver enthusiasm or caregivers’ belief that their child is interested in something, eliciting similar caregiver and subsequently child enthusiasm. While we found no overlap between caregiver perception of children’s interests and children’s looking behaviour in the category interest task, we found some evidence that caregivers were able to accurately estimate their child’s interests based on children’s overwhelming preference for the novel object from the high-interest book in the novel word-object recognition task and the different caregiver-centric and child-centric measures of QOI. Finally, while neither of these measures predicted learning, we found robust evidence that children learned the novel word-object associations from shared book reading. Thus, while the implications of the dynamics between caregivers and their children’s interests for learning remain inconclusive, the QOI between caregiver and child is clearly predicted by children’s interests and caregiver perception of children’s interests, and the nature of the joint task alone is sufficient to promote word learning. Perhaps, in this case, the journey—the increased quality of caregiver–child interaction during day-to-day proceedings—is the only reward.

## Data Availability

All data for this study (and corresponding analyses scripts) and study materials can be found in the OSF project page [89]. We cannot share the raw eye coding and video data to avoid breach of anonymity and data protection. Other statistics supporting this article have been uploaded as part of the supplementary material (also to be found in the OSF page) [102].

## Footnotes

^1^ The variable QOI was recalled between 0 and 1 using this formula: ((average of all 5 dimensions of the QOI score – 1)/5)
